# Reported Hydration Beliefs and Behaviors without Effect on Plasma Sodium in Endurance Athletes

**DOI:** 10.3389/fphys.2017.00259

**Published:** 2017-05-02

**Authors:** Daniela Chlíbková, Pantelis T. Nikolaidis, Thomas Rosemann, Beat Knechtle, Josef Bednář

**Affiliations:** ^1^Centre of Sports Activities, Brno University of TechnologyBrno, Czechia; ^2^Exercise Physiology LaboratoryNikaia, Greece; ^3^Institute of Primary Care, University of ZurichZurich, Switzerland; ^4^Gesundheitszentrum St. GallenSt. Gallen, Switzerland; ^5^Faculty of Mechanical Engineering, Brno University of TechnologyBrno, Czechia

**Keywords:** runners, mountain bikers, fluid intake

## Abstract

**Purpose:** Little information is available on the association of hydration beliefs and behaviors in endurance athletes and exercise-associated hyponatremia (EAH). The aim of the present study was to determine hydration beliefs and behaviors in endurance athletes.

**Method:** A 100 and 38 recreational athletes [107 mountain bikers (MTBers) and 31 runners] competing in seven different endurance and ultra-endurance races completed pre- and post-race questionnaires, and a subgroup of 113 (82%) participants (82 MTBers and 31 runners) also provided their blood samples.

**Result:** More than half of the participants had some pre-race (59%), mid-race (58%), and post-race (55%) drinking plan. However, the participants simultaneously reported that temperature (66%), thirst (52%), and plan (37%) affected their drinking behavior during the race. More experienced (years of active sport: *p* = 0.002; number of completed races: *p* < 0.026) and trained (*p* = 0.024) athletes with better race performance (*p* = 0.026) showed a more profound knowledge of EAH, nevertheless, this did not influence their planned hydration, reported fluid intake, or post-race plasma sodium. Thirteen (12%) hyponatremic participants did not differ in their hydration beliefs, race behaviors, or reported fluid intake from those without post-race EAH. Compared to MTBers, runners more often reported knowledge of the volumes of drinks offered at fluid stations (*p* < 0.001) and information on how much to drink pre-race (*p* < 0.001), yet this was not associated with having a drinking plan (*p* > 0.05). MTBers with hydration information planned more than other MTBers (*p* = 0.004). In comparison with runners, more MTBers reported riding with their own fluids (*p* < 0.001) and planning to drink at fluid stations (*p* = 0.003). On the whole, hydration information was positively associated with hydration planning (*n* = 138) (*p* = 0.003); nevertheless, the actual reported fluid intake did not differ between the group with and without hydration information, or with and without a pre-race drinking plan (*p* > 0.05).

**Conclusion:** In summary, hydration beliefs and behaviors in the endurance athletes do not appear to affect the development of asymptomatic EAH.

## Introduction

The main reason for fluid intake during endurance events is to reduce the fluid deficit caused by the loss of sweat (Garth and Burke, 2013). Guidelines have been developed and athletes are often advised to drink in order to replace body weight losses (Sawka et al., 2007) and prevent exertional heatstroke during prolonged exercise in the heat (Nolte et al., 2015). Some authors (Maughan and Shirreffs, 2008) claim that dehydration impairs both physical and mental performance. A large number of half-marathon and full-marathon runners (O'Neal et al., 2011) believe that dehydration results in major performance decrements and that heat-related symptoms are caused by inadequate fluid intake. Nevertheless, the increased participation of athletes in endurance and ultra-endurance races has led to the risk of exercise-associated hyponatremia (EAH) (Knoth et al., 2012). EAH occurring during or up to 24 h after physical activity is known to be a possible complication of endurance events and may have a fatal outcome (Noakes, 2012; Hew-Butler et al., 2015). The reported incidence of asymptomatic EAH ranges from 0 (Knechtle et al., 2011) to 51% (Lebus et al., 2010). The underlying pathophysiology of EAH involves overhydration in combination with fluid retention from nonosmotic stimulation of arginine vasopressin secretion (Bennett et al., 2014; Hew-Butler et al., 2015; Noakes et al., 2005). Excessive losses of urine sodium due to the secretion of brain natriuretic peptide may also contribute to the occurrence of EAH (Hew-Butler et al., 2008b). Therefore, there are also alternative views that *ad libitum* drinking is sufficient to replace sweat losses or that thirst should dictate the need for drinking (Noakes, 2011, 2012; Hew-Butler et al., 2015).

Drinking behavior is a complex entity (Phillips et al., 1984; Rolls, 1993; Armstrong et al., 2015) influenced by cultural factors, learned behaviors, distance to the source, and environmental conditions (Greenleaf, 1992; García-Rovés et al., 1998). Noakes (2012) and Smith et al. (1995) have suggested that genetic factors may also influence drinking behaviors during exercise and the identification of genetic markers that may predispose athletes to developing EAH is proposed for future research (Hew-Butler et al., 2015). Hydration behavior is a product of the belief systems and if pre-race beliefs have an effect on race behaviors, education may decrease the occurrence of EAH (Winger et al., 2011). Therefore, it seems logical that knowledge of the relationship between hydration beliefs and habits and plasma [Na^+^], education regarding proper hydration practices and organized educational programmes would be of great practical value to athletes and those working with them (e.g., coaches, sports nutritionists) (Nichols et al., 2005; Williams et al., 2012; Hew-Butler et al., 2015; Krabak et al., 2016).

Across the sparse literature on race hydration practices, there are examples of hydration behavior in endurance athletes. Based on the review by Garth and Burke (2013), a range of drinking strategies may be appropriate and athletes need to adopt an individualized approach to their hydration strategy. The survey of multi-stage mountain bikers by Rose et al. (2007) indicates that although more than half of the bikers did not acknowledge specific awareness of the official guidelines, over 80% reported drinking regularly during a race. Road cyclists during a 387-km race (Black et al., 2014) did not readily change their drinking behavior to match their sweat losses and were more at risk of EAH than of dehydration. Fluid over-consumption behaviors were evident during a running multi-stage ultra-marathon; however, water intake habits appeared to be sufficient to maintain euhydration state (Costa et al., 2013). Marathon runners lacked knowledge of fluid intake to prevent EAH during a race (Williams et al., 2012). Inexperienced runners are more likely to believe the “drink to stay ahead of thirst” dogma and according to Winger et al. (2011) curtailing this behavior could decrease the incidence of EAH. On the contrary, beliefs regarding the causes of EAH altered race behaviors of 161-km running race participants, but did not affect the development of EAH (Winger et al., 2013).

The primary purpose of our investigation was to determine if pre-race beliefs and race behaviors decrease the incidence of EAH amongst recreational endurance athletes. Therefore, we aimed (*i*) to determine hydration beliefs and behaviors of a joint group of endurance athletes (*n* = 138), (*ii*) to investigate hydration beliefs and drinking strategy between the groups of mountain bikers and runners, and (*iii*) to examine the association between hydration beliefs, drinking habits and the development of EAH by comparing hyponatremic and normonatremic groups.

## Materials and methods

### Ethical approval

The study was approved by the Ethics Committee of the Institute of Experimental Biology at Masaryk University, Brno, Czech Republic with written consent from all subjects. All subjects provided written consent in accordance with the Declaration of Helsinki. The protocol was approved by the ethics committee of the Institute of Experimental Biology at Masaryk University, Brno, Czech Republic.

### Data collection

We contacted all the participants in seven local running and mountain biking endurance and ultra-endurance races in the Czech Republic prior to the start of the study via e-mail and informed them about the investigation approximately 3 weeks before each race.

The “Czech Championship 24-h MTB race” took place on the second weekend of June in 2012 (12 testing subjects from a total of 91 participants) and 2013 (23 testing subjects from a total of 114 participants), the temperature range was 6–30° and 8–30°C, respectively and the average relative humidity was 43(1) and 44(2)%, respectively. The Bike Race Marathon Rohozec 24 h was held on June 9–10, 2012 (40 testing subjects from a total of 116 participants) [temperature range: 6–23°C, average relative humidity: 72(2)%]. The “Sri Chinmoy Self-Transcendence Marathon 24-h race” took place on July 21–22, 2012 (12 testing subjects from a total of 48 participants) [temperature range: 10–18°C, average relative humidity: 62(3)%]. The “Trilogy Mountain Bike Stage Race” was held in the first week of July in 2012 (14 testing subjects from a total of 206 participants) and 2013 (18 testing subjects from a total of 116 participants) [temperature range and average relative humidity: 22–33°C; 55(9)% and 12–26°C; 46(9)%, respectively] and the “Czech Championship 100-km running race” was held on March 9, 2013 (19 testing subjects from a total of 31 participants) [temperature range: −1–+1°C, average relative humidity: 65(4)%].

The athletes could consume beverages *ad libitum* from a buffet provided by the organizer offering warm and cold beverages, such as water, sports drinks, tea, soups, fruit juice, coke, coffee, and caffeinated drinks in each of the races. In 24-h races, there was one fluid station placed next to the circuit where participants deliver their bike or run performance. In 24-h mountain-bike races, the circuits measured 9.5 km (a single-track with an elevation of 220 m) and 12.6 km (a track with an elevation of 250 m), respectively. One lap of the 24-h running race measured 1 km (elevation of 1 m). There were approximately four fluid stations at every stage of the mountain-bike multi-stage race. (The first stage covered 66 km with 2,200 m of altitude to climb; the second stage was 63 km long with 2,300 m difference in elevation and the third stage was 78.8 km with 3,593 m change in altitude.) In the 100-km running race, there was one fluid station on a 1,500-m circuit without elevation. Further details on each race are provided elsewhere (Chlíbková et al., 2014a,b, 2015, 2016b). The data were collected over a total of seven races in 2012 and 2013.

All participants were invited to complete a web-based questionnaire prior to the race. A pilot study was conducted in 15 mountain bikers and 10 runners with a variable endurance experience. Questions which were found to be unclear were rephrased. The questionnaire consisted of several sections: demographic information including age, sex and pre-race experience (Table 1); pre-, mid- and post-race drinking strategies; planned type and volume of fluids to be consumed; source of information on fluid intake and knowledge of EAH (Table 2). Post-race questionnaires were distributed immediately after the race during blood sampling and the athletes provided information on their reported fluid intake during the race. Fluid intake was recorded by the athlete or by one of the members of the support team on a recording sheet during the race. At each fluid station, they marked the number of cups they consumed and the fluid intake was approximated by the support team. The athletes primarily ate and drank while running/cycling. We are aware of the fact that fluids from the fluid station could sometimes be used for other purposes than drinking (cooling, etc.). However, we assume that this is done predominantly near the fluid station, and the athletes with the members of the support team were asked to mark the number of actually consumed cups or bottles. The organizer did not provide the athletes with any special advice on the website regarding what and how much to drink during the race. Out of 145 volunteers who completed each of their races, 138 filled out pre- and post-race questionnaires.

**Table 1:** **Demographic, pre-race, and experience-related characteristics of all the finishers (***n*** = 138) in each type of race**.

**All finishers *n* = 138**	
**Age (y)**	37.9 (8.5)
**Male sex (%)**	80
**Years as an active biker/runner (y)**	9.1 (6.4)
**Number of similar completed races (*****n*****)**	8.3 (10.3)
**Total training hours per week (h)**	10.4 (4.5)
**Running/biking hours per week (h)**	9.4 (4.2)

*Data are reported as mean (SD), unless indicated as percentage. An average training week was based on the period of 3 months prior to the race*.

**Table 2:** **Percentage of answers regarding pre-, mid- and post-race drinking strategies; planned type and volume of fluids to be consumed; source of information on fluid intake and knowledge of EAH (***n*** = 138)**.

**Question**	**Answer**
“*Have you ever heard how much you should drink prior to, during and after the race?*”	53% “*Yes*”,47% “*No*” (single choice of two responses)
“*If so, where did you get the information?*”	27% “*Magazines*,” 15% “*Books*,” 65% “*Internet*,” 43% “*Friends*,” 1% “*Other*” (multiple responses)
“*Do you have any plan for your fluid intake before the race?*”	59% “*Yes*,”41% “*No*” (single choice of two responses)
“*If yes, how much*?” (with a specification 1 h before the race)	43% “*0.5L/h*,” 40% “*0.75L/h*,” 15% “*1L/h and more*” (2% of athletes did not report the amount of fluids.)
“*Do you have any plan for your fluid intake during the race?*”	58% “*Yes*,” 42% “*No*” (single choice of two responses)
“*If yes, how much*?” (with a specification during the whole race)	14% “*0.5L/h*,” 3% “*0.75L/h,” 83*% “*1L/h and more*”
“*Do you have any plan for your fluid intake after the race?*”	55% “*Yes*,” 45% “*No*” (single choice of two responses)
“*If yes, how much*?” (with a specification 1 h after the race)	26% “*0.5L/h*,” 33% “*0.75L/h*,” 36% “*1L/h and more*” (5% of athletes did not report the amount of fluids.)
“*What affects your drinking strategy during the race?*”	66% “*Temperature*,” 37% “*My plan*,” 52% “*Thirst*,” 6% “*Nothing*,”11% “*Other*” (multiple responses)
“*Have you ever heard about hyponatremia?*”	30% “*Yes, I know what it is*,” 15% “*Yes, but I do not know anything about it*,” 55% “*No*” (single choice of three responses)
“*Do you know the causes and consequences of hyponatremia?*”	12% “*Yes, I know the causes and consequences*,” 7% “*I only know the consequences*,”81% “*No*” (single choice of three responses)
“*Do you drink only sports drinks, just water or both?*”	23% “*Sports drinks*”, 4% “*Water*,” 67% “*Both*” (single choice of three responses) (6% of athletes did not provide any information)
“*Do you plan to drink at the fluid station during the race?*”	23% “*Yes*,” 54% “*Often*,” 23% “*No*” (single choice of three responses)
“*Do you know the volume of the drinks which are offered at the fluid station?*”	70% “*Yes*,”30% “*No*” (single choice of two responses)
“*Do you run/ride with your own fluids?*”	4% “*Yes*,”9% “*Sometimes*,” 87% “*No*” (single choice of three responses)

Blood samples were collected from the antecubital vein prior to the race and immediately after crossing the finishing line according to the previously described methods (Chlíbková et al., 2014a, 2015, 2016b). Blood samples were obtained in order to determine pre- and post-race plasma [Na^+^] on a Roche Modular Analytics (SWA), Modules P& ISE (Hitachi High Technologies Corporation, Japan, Roche Diagnostic). The athletes could drink before the race and during the race without any intervention.

### Statistical analysis

All data were tested for normality using the Kolmogorov-Smirnov test and were presented as mean ± standard deviation. The data of the participants were analyzed using unpaired *t-*tests, binary logistic regression (continuous data stratified by a categorical factor), Kruskal-Wallis test (for comparing two or more independent samples of equal or different sample sizes), Chi-squared test (test of independence of two categorical variables), Fisher's exact test (test of independence of two categorical variables with two levels), and the Spearman rank-order correlation (test of independence of two ordinal variables). Data regarding race performance were obtained from the website of each race. Race performance was calculated as relative place in each race in percentiles. The significance level was set at *p* < 0.05. All statistical analyses were performed using the MINITAB (Version 17.2; Minitab, Inc, USA) and STATISTICA (Version 12.0; StatSoft, Tulsa, OK, USA).

## Results

### Sample characteristics

One hundred and forty-five endurance athletes participated in this study. A total of 138 of them (95%) (Twelve 24-h runners, seventy-five 24-h mountain bikers, 32 multi-stage mountain bikers, and nineteen 100-km runners) completed the pre-and post-race questionnaires. A subgroup of 113 (82%) volunteers (88 men and 25 women including twelve 24-h runners, fifty 24-h mountain bikers, 32 multi-stage mountain bikers, and nineteen 100-km runners) provided pre- and post-race blood samples. EAH is defined as a serum, plasma or blood [Na^+^] concentration below the normal reference laboratory range of [Na^+^], i.e., <135 mmol/L (Hew-Butler et al., 2008a). Thirteen finishers (12%) were found to have mild post-race EAH (plasma [Na^+^] ranging between 129 and 134 mmol/L) (Chlíbková et al., 2016b), corresponding to 16% runners and 10% mountain bikers. No differences in pre-race characteristics, such as age, sex and pre-race experience, were observed among the participants in all types of races; the groups were homogeneous (Chlíbková et al., 2016a). The number of years of active running/biking, number of similar completed races, total training hours per week and the number of running/biking hours per week (*n* = 138) are listed in Table 1. We found no association between age, sex, years of practicing an active sport or the number of completed races and groups of athletes with or without a pre-, mid- or post-race drinking plan (*p* > 0.05), or endurance athletes grouped by the reported influence of plan, thirst or temperature on their drinking during a race (*p* > 0.05).

### Information about hydration and its sources; hydration plans for the period before, during and after each race

The percentage of answers regarding information on hydration, sources of information and planned fluid intake from all participants (*n* = 138) and from the athletes with EAH (*n* = 13) and without EAH (*n* = 100) together with their statistical comparison is shown in Tables 2, 3, respectively. An affirmative answer to the question: “*Have you ever heard how much you should drink prior to, during and after the race?*” is positively associated with an affirmative answer to the question: “*Do you have any plan for your fluid intake during the race?*” (*p* = 0.003).

**Table 3:** **Percentage of answers regarding pre-, mid- and post-race drinking strategies; planned type and volume of fluids to be consumed; source of information on fluid intake and knowledge of EAH in the hyponatremic (EAH) (***n*** = 13) and normonatremic (non-EAH) (***n*** = 100) groups**.

**Question**	**Answer**	**Fisher's exact test (EAH vs. non-EAH)**
“*Have you ever heard how much you should drink prior to, during and after the race?*”	69% EAH and 56% non-EAH “*Yes*” 31% EAH and 44% non-EAH “*No*”	*p* = 0.552
“*If so, where did you get the information?*”	23% EAH and 16% non-EAH “*Magazines*” 0% EAH and 9% non-EAH “*Books*” 31% EAH and 38% non-EAH “*Internet*” 39% EAH and 22% non-EAH “*Friends*” 0% EAH and 1% non-EAH “*Other*”	Not compared^*^
“*Do you have any plan for your fluid intake before the race?*”	69% EAH and 59% non-EAH “*Yes*” 31% EAH and 41% non-EAH “*No*”	*p* = 0.559
“*If yes, how much*?” (with a specification 1 h before the race)	56% EAH and 47% non-EAH “*0.5L/h*” 44% EAH and 36% non- EAH “*0.75L/h*” 0% EAH and 17% non-EAH “*1L/h and more*”	*p* = 0.730
“*Do you have any plan for your fluid intake during the race?*”	54% EAH and 59% non-EAH “*Yes*” 46% EAH and 41% non-EAH “*No*” (single choice of two responses)	*p* = 0.771
“*If yes, how much*?” (with a specification during the whole race)	14% EAH and 13% non-EAH “*0.5L/h*”, 14% EAH and 5% non-EAH “*0.75L/h*” 72% EAH and 82% non-EAH “*1L/h and more*”	Not compared^*^
“*Do you have any plan for your fluid intake after the race?*”	69% EAH and 57% non-EAH “*Yes*” 31% EAH and 43% non-EAH “*No*”	*p* = 0.553
“*If yes, how much*?” (with a specification 1 h after the race)	22% EAH and 28% non-EAH “*0.5L/h*” 33% EAH and 32% non-EAH “*0.75L/h*” 45% EAH and 35% non-EAH “*1L/h and more*” (5% of non-EAH athletes did not report the amount of fluids.)	Not compared^*^
“*What affects your drinking strategy during the race?*”	69% EAH and 65% non-EAH “*Temperature*” 31% EAH and 34% non-EAH “*Own schedule*” 54% EAH and 56% non-EAH “*Thirst*” 15% EAH and 6% non-EAH “*Nothing*” 8% EAH and 13% non-EAH “*Other*”	Not compared^*^
“*Have you ever heard about hyponatremia?*”	23% EAH and 59% non-EAH “*Yes, I know what it is*” 8% EAH and 11% non-EAH “*Yes, but I do not know anything about it*” 69% EAH and 30% non-EAH “*No*”	*p* = 0.137 ^**^
“*Do you know the causes and consequences of hyponatremia?*”	8% EAH and 11% non-EAH % “*Yes, I know the causes and consequences*” 8% EAH and 11% non-EAH “*I only know the consequences*” 84% EAH and 78% non-EAH “*No*”	*p* = 0.732 ^**^
“*Do you drink only sports drinks, just water or both?*”	31% EAH and 22% non-EAH “*Sports drinks*,” 15% EAH and 4% non-EAH “*Water*,” 54% EAH and 74% non-EAH “*Both*”	Not compared^*^
“*Do you plan to drink at the fluid station during the race?*”	23% EAH and 26% non-EAH “*Yes*” 39% EAH and 54% non-EAH “*Often*” 39% EAH and 20% non-EAH “*No*”	Not compared^*^
“*Do you know the volume of the drinks which are offered at the fluid station?*”	23% EAH and 34% non-EAH “*Yes*” 77% EAH and 66% non-EAH “*No*”	Not compared^*^
“*Do you run/ride with your own fluids?*”	0% EAH and 5% non-EAH “*Yes*” 0% EAH and 8% non-EAH “*Sometimes*” 100% EAH and 87% non-EAH “*No*”	Not compared^*^

The differences between the EAH and non-EAH groups were calculated using the Fisher's exact test. Not compared^*^, The EAH and the non-EAH groups were not compared, as there was not enough data in each subgroup for statistical comparison. The observed categorical variables have more than two levels and grouping was not appropriate.

** *, In our statistical analysis, both affirmative answers were combined to increase the number of respondents in each group*.

### Plans for drinking before the race

The differences in answers regarding the planned fluid intake between the hyponatremic and normonatremic groups (Table 3) were not significant (*p* > 0.05). 64 (60%) mountain-bikers and 18 (58%) runners had a plan for fluid intake before the race, without significant differences between the two groups (*p* < 0.05).

### Plans for drinking during the race

The differences in answers regarding plans for drinking during the race between the hyponatremic and normonatremic groups (Table 3) were not significant (*p* > 0.05). The distribution of the answers of mountain-bikers and runners to questions: “*Have you ever heard how much you should drink prior to, during and after the race?*” and “*Do you have any plan for your fluid intake during the race?*” is shown in Table 4. Compared to mountain bikers, runners significantly more often reported that they had heard about how much to drink (*p* < 0.001), yet this was not associated with having a drinking plan during the race (*p* > 0.05). Mountain bikers who had pre-race information on how much to drink planned their hydration more frequently than those without the information (*p* = 0.004). The differences in answers regarding the planned fluid intake during the race between mountain bikers and runners were not significant (*p* > 0.05). Using a binary logistic regression test, no association between post-race plasma [Na^+^] and the groups of athletes with or without drinking plan during a race was found (*p* > 0.05).

**Table 4:** **Distribution of the answers of mountain-bikers and runners to questionnaire questions: “***Have you ever heard how much you should drink prior to, during and after the race?***” and “***Do you have any plan for your fluid intake during the race?***”**.

**Mountain-bikers (*n* = 107)**	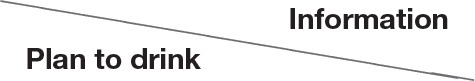	**Yes**	**No**	∑
	Yes	36 (34%)	28 (26%)	64 (60%)
	No	12 (11%)	31 (29%)	43 (40%)
	∑	48 (45%)	59 (55%)	107 (100%)
**Runners (*****n*** = **31)**	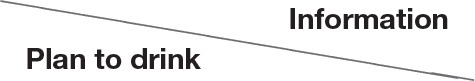	**Yes**	**No**	∑
	Yes	15 (48%)	3 (10%)	18 (58%)
	No	10 (32%)	3 (10%)	13 (42%)
	∑	25 (80%)	6 (20%)	31 (100%)

*Information, “Have you ever heard how much you should drink prior to, during and after the race?.” Plan to drink, “Do you have any plan for your fluid intake during the race?.” The number of athletes is expressed in absolute values and as a percentage of the number of athletes in the individual disciplines in brackets*.

### Plans for drinking after the race

The differences in answers regarding plans for drinking after the race between the hyponatremic and normonatremic groups (Table 3) were not significant (*p* > 0.05). 58 (54%) mountain-bikers and 17 (55%) runners had a plan for fluid intake after the race, without significant differences between the two groups (*p* > 0.05).

### Drinking behaviors and habits

The percentage of answers regarding drinking behaviors and habits from all the participants (*n* = 138) and from the athletes with EAH (*n* = 13) and without EAH (*n* = 100) is shown in Tables 2, 3, respectively. When comparing mountain bikers and runners, mountain bikers more often reported carrying their own fluids (mountain-bikers: 13%, runners: 0%) (*p* < 0.001) and drinking at fluid stations (mountain-bikers: 25%, runners: 16%) (*p* = 0.003). On the contrary, runners reported a higher knowledge of the volumes offered at fluid stations (mountain-bikers: 22%, runners: 61%) (*p* < 0.001). Mountain bikers (70%) and runners (80%) reported drinking both sports drinks and water, without significant differences between the two groups (*p* > 0.05). The differences in answers regarding drinking behaviors and habits between the EAH and non-EAH groups were not compared, as there was not enough data in each subgroup for statistical comparison.

### Influences on drinking behavior during the race

The percentage of answers from all participants (*n* = 138) and from the athletes with EAH (*n* = 13) and without EAH (*n* = 100) regarding the influences on their drinking behaviors during the race is shown in Tables 2, 3, respectively. Fifty-four (68%) athletes from the group with a plan for drinking during the race reported “*Temperature*,” forty (50%) reported “*My plan*” and thirty-eight (48%) selected “*Thirst.*” Thirty-seven (64%) athletes from the group without a plan reported “*Temperature*” and 34 (59%) mentioned “*Thirst*” (multiple choice). “*Temperature*” was the most commonly selected answer from the options that influence drinking strategy during the race (63% of mountain-bikers and 77% of runners). The EAH and non-EAH groups were not compared, as there was not enough data regarding the influences on their drinking behaviors during a race for statistical comparison.

### Reported fluid intake

Post-race reported fluid intake during the race and pre-race planned fluid intake with the distribution of all athletes, mountain bikers and runners is shown in Table 5. On average, the reported fluid intake during the race was 0.55 (0.3) L/h (*n* = 138); 0.68 (0.3) L/h in the hyponatremic and 0.56 (0.3) L/h in the normonatremic groups; 0.53 (0.2) L/h in mountain bikers and 0.62 (0.4) L/h in runners. The individual amounts of fluid intake in hyponatremic subjects are shown in Table 6. Participants with a plan for drinking drank an average of 0.55 (0.3) L/h; their median intake of fluids was 0.50 L/h (IQR 0.40–0.73 L/h). Athletes without the plan drank an average of 0.56 (0.3) L/h; their median intake of fluids during the race was 0.50 L/h (IQR 0.40–0.75 L/h). Neither the information on how much to drink during the race, nor having a drinking plan had any significant association with the reported fluid intake (*p* > 0.05). We found no association between the reported fluid intake and post-race plasma [Na^+^], absolute or percentage change of plasma [Na^+^]. The place in the race was not associated with the reported fluid intake (*p* > 0.05) (*n* = 138).

**Table 5:** **Post-race reported fluid intake in the post-race questionnaire and pre-race planned fluid intake in the pre-race questionnaire (***n*** = 138)**.

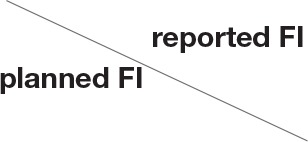	**0.1 – 0.5 L/h**	**0.6 – 0.75 L/h**	**0.76 – < 1.0 L/h**
	**Total (MTB, R); percentage values**	**Total (MTB, R); percentage values**	**Total (MTB, R); percentage values**
0.5 L/h	7 (7, 0); 5% (7%, 0%)	3 (3, 0); 2% (3%, 0%)	(0, 0); 0% (0%, 0%)
0.75 L/h	1 (0, 1); 1% (0%, 3%)	1 (0, 1); 1% (0%, 3%)	(0, 0); 0% (0%, 0%)
1 L/h and more	39 (28, 11); 28% (26%, 36%)	15 (12, 3); 11% (11%, 10%)	12 (7, 5); 9% (6%, 16%)
No plan	35 (32, 3); 25% (30%, 9%)	16 (14, 2); 11% (13%, 6%)	9 (4, 5); 6% (4%, 16%)
∑	82 (67, 15); 59% (63%, 48%)	35 (29, 6); 25% (27%, 19%)	21 (11, 10); 15% (10%, 32%)

*Reported FI, post-race reported fluid intake in the post-race questionnaire; planned FI, pre-race planned fluid intake in pre-race questionnaire (n = 138). Total, total number of all participants. The proportion of mountain bikers and runners is expressed in brackets. MTB, mountain-bikers, R, runners*.

**Table 6:** **Finishers with EAH and their demographic and pre-race training characteristics, place in the race, pre- and post-race plasma [Na^**+**^] concentration, planned and actual reported fluid intake during the race**.

**EAH cases**	**1**	**2**	**3**	**4**	**5**	**6**	**7**	**8**	**9**	**10**	**11**	**12**	**13**
Gender	M	F	M	M	M	M	F	F	M	M	M	F	M
Age (years)	39	38	42	40	33	26	46	33	51	48	35	48	35
Race (discipline)	24 km MTB	24 km RUN	MTB stage	24 km MTB	24 km MTB	24 km MTB	24 km MTB	24 km MTB	100 km RUN	100 km RUN	100 km RUN	100 km RUN	MTB stage
Number of completed ultra-marathons (n)	3	30	0	6	5	5	11	5	4	3	17	40	4
Years as an active biker/runner	3	13	5	4	9	7	11	4	27	10	13	8	8
Relative place in the race in percentiles (%)	0.08	0.02	0.77	0.09	0.02	0.39	0.14	0.30	0.61	0.35	0.94	0.52	0.81
Pre-race blood [Na^+^]	138	137	142	138	138	136	139	137	138	142	142	141	141
Post-race blood [Na^+^]	129	133	134	134	134	132	134	134	134	134	134	134	133
Planned fluid intake (no plan, 0.5 L/h, 0.75 L/h, 1 L/h and >1 L/h)	>1	no plan	0.50	>1	no plan	1	no plan	no plan	no plan	1	1	0.75	no plan
Reported fluid intake (L/h)	1.0	0.8	0.8	0.5	0.4	0.7	0.6	0.6	0.8	1.5	0.2	0.4	0.8

*MTB, mountain biking; RUN, running*.

### Athletes' knowledge of hyponatremia

**Figure 1:**
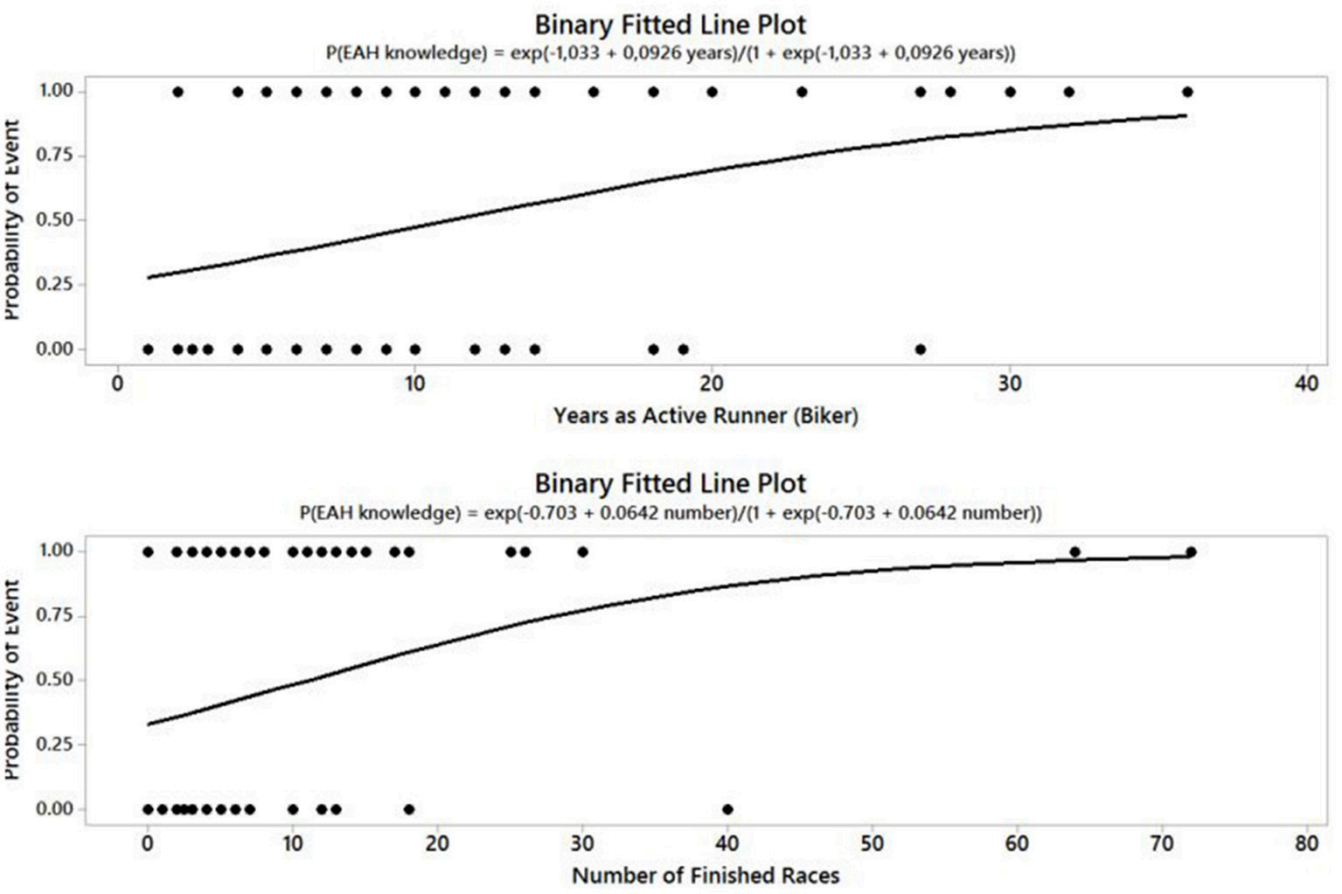
**Probability model of reported affirmative answers to questions on the knowledge of EAH (***n*** = 138) considering the number of years as an active runner/biker (***p*** = 0.002) and the number of similar completed races (***p*** < 0.001)**.

The reported knowledge of EAH (Table 2) was associated with the number of years of active running/biking (*p* = 0.002), number of similar completed races (*p* < 0.001), total training hours per week (*p* = 0.024) and the relative place in the race (*p* = 0.026) (Figure 1). These associations were tested by binary logistic regression. In our statistical analysis, we combined both affirmative answers to increase the number of respondents in each group. 50% of mountain-bikers and 29% of runners gave an affirmative answer to the question “*Have you ever heard about hyponatremia?.*” There were no significant differences in answers between the EAH and non-EAH groups (Table 3). The reported knowledge of EAH was not associated with having or not having a plan for drinking during the race (*p* > 0.05), reported fluid intake during the race (*p* > 0.05), pre-race information about how much to drink (*p* > 0.05), influences on the drinking plan during the race (*p* > 0.05), or post-race plasma [Na^+^].

## Discussion

The most important finding was that the hyponatremic and normonatremic endurance athletes in our study did not differ in their reported hydration beliefs, behaviors and fluid intake during the race. Hydration beliefs and behaviors in these athletes do not appear to affect the development of EAH for reasons that remain unclear.

More than half of the athletes, with no differences between the EAH and non-EAH groups, reported that they had some information on how much to drink prior to, during and after the race and that they had a plan for their fluid intake before, during and after the race. That is a considerably lower proportion in comparison with the 42-km marathoners in the study by Williams et al. (2012). Nonetheless, we found a positive association between having hydration information and pre-race hydration planning among all participants; however, without influence on the actual fluid intake reported post-race. With regard to sports discipline, runners reported having heard about how much to drink more often than mountain bikers; nevertheless, without any relation to having a plan for drinking during the race. On the contrary, mountain bikers with this information planned more than those without the information. On the whole, however, planned hydration did not differ between mountain bikers and runners.

The athletes in our study were mainly influenced by the internet, followed by information from their friends and magazines. According to the study by Winger et al. (2011), runners most commonly reported personal experience, followed by recommendations from their friends. Half-marathon and full-marathon runners preferred interpersonal contacts with other runners to information from literature, professional association position stands, or advertisements (O'Neal et al., 2011). Williams et al. (2012) reported the London Marathon magazine as the prime source of knowledge, followed by information from friends with running experience. Multi-stage mountain bikers described in the study by Rose et al. (2007) based their approach to fluid intake on their own personal experience, followed by advice from friends and magazines. In the present study, the least amount of fluid intake was planned by athletes who obtained information on hydration primarily by reading magazines, followed by information from friends. By contrast, the highest amount of fluid intake was planned by those who were influenced by the internet. When comparing athletes with EAH and without EAH, the internet prevailed in normonatremic athletes, whereas information from friends was more important in hyponatremic athletes. The internet appears to have played an important role in the athletes' hydration beliefs and served as an important source of information.

Participants with a plan, both hyponatremic and normonatremic athletes in both disciplines, most often reported drinking 1 L/h or more during and after the race. Fluid intake during single-day ultra-endurance events in which the top competitors finish in > 3 h ranges from 300 to 1000 mL/h (Garth and Burke, 2013) with the incidence of EAH in 0–51% of the study participants. A vast majority of the 42-km marathon runners had some information on drinking strategy; nevertheless, some of them planned to drink more than 3.5 L, which could put them at greater risk of EAH according to the authors (Williams et al., 2012). Runners exhibit behavior that is shaped by their beliefs about the benefits and risks of hydration (Winger et al., 2013). It is worth noting that nearly 9% of runners planned to drink as much as possible during the race and training in the survey by Winger et al. (2011), as well as 12% in the study by Williams et al. (2012). As noted by Black et al. (2014), sixteen out of eighteen road cyclists in a 387-km cycling race reported post-race that they had drunk as much as was tolerable in order to prevent dehydration, with an average intake of 0.58 L/h. Road cyclists in a 164-km cycling race reported an average intake of 0.70 L/h (Armstrong et al., 2015). About half of the multi-stage mountain bikers in the study by Rose et al. (2007) reported pre-race that they planned to drink <0.75 L/h; 45% of the bikers had a planned fluid between 0.75 and 1.50 L/h and 8% of them more than 1.50 L/h. Nevertheless, when comparing fluid intake in cyclists with pre-race questionnaires and in those with questionnaires after the first 2 days of the race in the present study, most cyclists in the latter group mentioned <0.75 L/h and reported post-race that their average fluid intake during the race was 0.55 (0.3) L/h (Rose et al., 2007). When comparing the actual fluid intake during the race, most of the present participants failed to follow their plan and drank less during the race, with an average of 0.37 L/h (0.3). Racing speed determines metabolic rate and sweat rate, as well as fluid replacement needs (Noakes, 2012). Interestingly, in the present study, athletes with better race performance or higher number of active years as a runner/biker did not report higher or lower fluid intake than other finishers. The above-mentioned findings suggest that circumstances in the actual race prevail over the athletes' pre-race decision to have or not to have a drinking plan. This presumption was supported by the answers to the question “*What affects your drinking strategy during the race?*” with temperature being cited most often in both the group with and without a plan, followed by thirst and plan with nearly the same number of respondents in the group with a plan. Hydration strategies varied greatly between individuals and they are also influenced by immediate environmental conditions and other factors (Maughan and Shirreffs, 2008). It may seem paradoxical that the athletes in the present study reported post-race that their fluid intake was lower than planned, especially given that temperature was the most frequently reported influence on their drinking strategy during the race. We interpret this to mean that factors other than temperature and thirst, such as the overestimation of their personal fluid intake plan elaborated based on advice from the internet where we can often find very different sources of information, were involved during their pre-race planning. Commercial influence might delay the acceptance of certain scientific findings (Noakes, 2011). Therefore, it seems that the present athletes naturally reduced their planned fluid intake during the race based on ambient temperature and thirst during each race.

Thirst was the second most frequently reported influence on fluid intake during the race. Drinking to thirst has been demonstrated in many studies to be safe and without any detrimental effect on performance (Noakes et al., 2005; Noakes, 2012; Hoffman and Stuempfle, 2014). In the study by Hoffman and Stuempfle (2014), drinking to thirst was the most common type of drinking in 161-km ultra-marathoners. A total of 58% of runners from three races in 2009 drank only when thirsty during the race (Hoffman and Stuempfle, 2014). Ultra-marathoners who drank to thirst in a 82-km running race consumed smaller fluid volumes and had higher post-race plasma [Na^+^] than those with a pre-planned hydration protocol (Scotney and Reid, 2015). On the contrary, in the survey by Winger et al. (2013) investigating 161-km ultra-marathon runners, only 17% of the runners reported drinking to thirst for unclear reasons and 44% planned to drink according to a predetermined schedule. Similarly, only 25% of the 42-km marathon runners identified thirst as the most important factor determining their fluid intake (Williams et al., 2012). We assume that the present athletes could also adjust their pre-race planned fluid intake according to their physiological needs to decrease fluid consumption (increased urination, bloating, weight gain) while running or biking and were flexible with respect to their needs. Further studies of real-life hydration practices including information on motives for drinking or not (Garth and Burke, 2013) and gathering evidence with regards to the success of the “drink to thirst” strategy in the prevention of EAH is one of the remaining issues for future research studies (Hew-Butler et al., 2015).

According to Armstrong et al. (2014, 2016), runners and cyclists have different drinking beliefs due to the different availability of fluids during running and cycling. Differences between the performance of mountain bikers, road cyclists and runners would favor a more positive fluid balance in cyclists compared to runners (Scotney and Reid, 2015). With regard to other drinking behaviors and habits, most of the endurance athletes in the present study drank a combination of water and sports drinks, similarly to endurance runners in the study by Winger et al. (2011), marathoners in the study by Williams et al. (2012) and multi-stage mountain bikers in the study by Rose et al. (2007). Although compared to runners, mountain-bikers in the present study significantly more often reported carrying own fluids, planned to consume fluids at fluid stations during the race, had a significantly lower knowledge of the volumes of fluids offered at fluid stations, their planned and reported fluid intake was not significantly different from the group of runners.

More than half of the athletes in the present study reported no knowledge of EAH and a majority of them reported no knowledge of the causes and consequences of EAH. Similarly, 65% of the 42-km marathon runners had heard about EAH; however, only 37% had knowledge of its causes and effects in the study by Williams et al. (2012). Also, 89% of multi-stage mountain bikers (Rose et al., 2007) felt a need for more education concerning fluid intake. However, no association between post-race plasma [Na^+^], reported fluid intake and knowledge of EAH has been found in the present study, with no differences between the normonatremic and hyponatremic athletes. This fact was consistent with the findings of Winger et al. (2011) with no differences in post-race plasma [Na^+^] in runners and different levels of knowledge regarding fluid physiology. One may assume that experienced endurance athletes are well trained and know their personal needs during an endurance race. In our study, more experienced and trained participants with better place in the race reported knowledge of EAH more frequently, with no difference between mountain bikers and runners; yet without any influence on their hydration beliefs, behaviors, fluid intake, or post-race plasma [Na^+^]. Moreover, pre-race characteristics, such as the level of experience, had no influence on the hydration strategy and fluid intake during the race, as opposed to the findings by Winger et al. (2011).

## Limitations

The study was limited by the usual difficulties of self-reported survey data and possible discrepancies in reports due to tiredness. Given the field nature of this survey, self-reported fluid intakes may be inaccurate. Another limitation concerned the number of endurance athletes in various disciplines and sex representation due to the nature and toughness of such races. It may not be possible to recruit sufficient number of participants given the very nature of this type of races. Moreover, there was the possibility of one athlete participating on multiple occasions and that prior knowledge of the questionnaire could influence their results. However, the disciplines were very different in the two sports and this possibility could only occur in the data from races which were monitored for two consecutive years. This was the case in about 3% of the athletes (normonatremic), which was deemed to have a small influence on our findings. Future studies should investigate a larger cohort of endurance athletes. It was not possible to compare all answers between hyponatremic and normonatremic athletes due to the small number of answers in the hyponatremic group.

## Conclusions

More experienced and trained participants with better place in the race showed a higher knowledge of EAH, which, however, did not influence their hydration strategy, or their post-race plasma [Na^+^]. Hydration information was positively associated with hydration planning; nevertheless, the actual reported fluid intake did not differ between the groups with and without hydration information or with and without a pre-race drinking plan. Asymptomatic hyponatremic participants did not differ in hydration beliefs, race behaviors or reported fluid intake from those without post-race EAH. This could be the reason why post-race plasma [Na^+^] was not determined by the athletes' pre-race planning. In summary, hydration beliefs and behaviors in the present endurance athletes do not appear to affect the development of EAH. More studies are needed to determine if information about EAH could influence its prevalence.

## Author contributions

DC designed the study, collected all data and drafted the manuscript, PN, TR, and BK helped in designing the study and drafting the manuscript, JB performed the statistical analyses.

### Conflict of interest statement

The authors declare that the research was conducted in the absence of any commercial or financial relationships that could be construed as a potential conflict of interest.
